# Supplementary material for: Clonal Amplification-Enhanced Gene Expression in Synthetic Vesicles
Source: ACS Synth Biol. 2023 Apr 4;12(4):1187–203. doi: 10.1021/acssynbio.2c00668 (PMC10127449; doi:10.1021/acssynbio.2c00668)
Supplement: Supplementary file 1 — sb2c00668_si_001.pdf [file sb2c00668_si_001.pdf]

SUPPLEMENTARY INFORMATION

Clonal amplification-enhanced gene expression in synthetic vesicles

Zhanar Abil[1,†], Ana Maria Restrepo Sierra[1,†], Christophe Danelon[1,2,*]

[1]Department of Bionanoscience, Kavli Institute of Nanoscience, Delft University of Technology, 2629HZ Delft, The Netherlands

[2]Toulouse Biotechnology Institute (TBI), Université de Toulouse, CNRS, INRAE, INSA, 31077 Toulouse, France

[†]Equal contribution

*Correspondence, Email: c.j.a.danelon@tudelft.nl or danelon@insa-toulouse.fr

Content:

Page 2 | **Table S1.** Estimated protein production rate from LC-MS data.

Page 2 | **Table S2.** Enrichment efficiency of ori-*yfp*.

Page 2 | **Table S3.** Probability of having one or more DNA molecules per liposome, $P(k \geq 1)$.

Page 3 | **Table S4.** DNA primer sequences and purpose.

Page 3 | **Table S5.** Plasmid DNA description.

Pages 4-6 | **Table S6.** Transitions of the MS/MS measurements for the proteolytic peptides of the indicated proteins.

Page 7 | **Fig S1.** Relative protein quantification from bulk reactions with and without expCADGE.

Page 7 | **Fig S2.** LC-MS raw data peaks of the YFP and PssA proteolytic peptides used for relative quantification.

Page 8 | **Fig S3.** Effect of DNA concentration on YFP protein expression without gene amplification.

Page 8 | **Fig S4.** End-point YFP fluorescence measurements from ori-*yfp* bulk IVTT reactions.

Page 9 | **Fig S5.** Individual qPCR data from Figure 4h.

Page 9 | **Fig S6.** Raw FACS data of liposome samples with appended gating line as used in Figure 5c,d.

Page 10 | **Fig S7.** In-liposome expression of ori-*pssA* under different DNA concentrations (10 nM to 10 pM) without DNA replication.

Page 10 | **Fig S8.** FACS data of liposome samples analyzed in Figure 5g.

Page 11 | **Fig S9.** Liposomes FACS data and gating strategy for Figure 5h.

Page 12 | **Fig S10.** Quantitative analysis of liposome size distribution and DNA occupancy.

Page 13 | **Fig S11.** Data processing for FACS data.

Supplementary Table 1. Estimated protein production rate from LC-MS data.

CADGE experimental condition	Estimated production rate
PssA expCADGE	6.4 nM/min
PssA purCADGE	5.3 nM/min
YFP expCADGE	3.8 nM/min
YFP purCADGE	4.1 nM/min

The highest slope of the curve was calculated.

Supplementary Table 2. Enrichment efficiency of ori-*yfp*.

Experimental condition	Enrichment efficiency
IVTT (all gate sorting)	31.51
expCADGE (all gate sorting)	13.24
expCADGE (high gate sorting)	40.43
purCADGE (all gate sorting)	17.32
purCADGE (high gate sorting)	89.69

Supplementary Table 3. Probability of having one or more DNA molecules per liposome, $P(k \geq 1)$.

	$P\ (k \geq 1)$	
**Sample type**	***d* is fixed (mean)**	***d* is distributed**
YFP (Fig 2)	0.214	0.248
YFP (Fig 2)	0.169	0.211
PssA (Fig 5)	0.094	0.125
MinD (Fig 6)	0.246	0.274
MinD (Fig 6)	0.261	0.288

Figure numbers refer to the figures in the main text. Values for the diameter (*d*) are derived from the data in Fig. S10.

Supplementary Table 4. DNA primer sequences and purpose.

Name	Purpose	Sequence	Modifications
1106 ChD	YFP gene subcloning	CCGTTTAGAGGCCCCAAGGG	none
1107 ChD	YFP gene subcloning	CTTCGTCTGTGTCGCATGTGAaATTAATACGACTCACTATAGGGAGACCACAACG	none
1104 ChD	amplification of vector	TAGCATAACCCCTTGGGGC	none
1105 ChD	amplification of vector	CCTATAGTGAGTCGTATTAATtTCACATGCGAC	none
1115 ChD	pssA gene subcloning	CTTCGTCTGTGTCGCATGTGAaATTAATACGACTCACTATAGGGGAATTGTGAGC	none
1116 ChD	pssA gene subcloning	AACCCCTCAAGACCCGTTTAGAG	none
961 ChD	TP gene cloning	acgtggtaccAAAGTAAGCCCCCACCCTCACATG	none
962 ChD	TP gene cloning	agctaagcttAAAGTAGGGTACAGCGACAACATACAC	none
491 ChD	preparative PCR for IVTTR	5-phos/AAAGTAAGCCCCCACCCTCACATG	5'-phosphorylation
492 ChD	preparative PCR for IVTTR	5-phos/AAAGTAGGGTACAGCGACAACATACAC	5'-phosphorylation
1121 ChD	YFP detection	TGCAACTGGCTGACCACTAC	none
1122 ChD	YFP detection	AATGATTGTCCGGCAGCAGA	none
980 ChD	p3 detection	ACGGCTGAAATTGACATCCCG	none
981 ChD	p3 detection	CCAGGCGTTGAACTTCTTTGG	none
1125 ChD	pssA-qPCR-F	AACAGGATGACGGTGGCAAA	none
1126 ChD	pssA-qPCR-R	GGAACATCTACGCCCGGATT	none
1208 ChD	MinD detection	CGCGACTCTGACCGTATTT	none
1209 ChD	MinD detection	AGCATGTCACCTCTGCTTAC	none

Supplementary Table 5. Plasmid DNA description.

plasmid name	plasmid description
**G365**	Contains the DNA unit for the expression of YFP fluorescence protein. Transcription is regulated by a T7 promoter and T7 terminator sequences. The entire CDS unit is placed in between right and left origins of replication from the Φ29 DNA replication machinery.
**G368**	Contains the DNA unit for the expression of the phospholipid biosynthesis protein PssA. Transcription is regulated by a T7 promoter and T7 terminator sequences. The entire CDS unit is placed in between right and left origins of replication from the Φ29 DNA replication machinery.
**G437**	Contains the DNA unit for the expression of MinD protein. Transcription is regulated by a T7 promoter and T7 terminator sequences. The entire CDS unit is placed in between right and left origins of replication from the Φ29 DNA replication machinery.
**G338**	Contains the DNA unit for the expression of the Φ29 terminal protein TP. Transcription is regulated by a T7 promoter and T7 terminator sequences. The entire CDS unit is placed in between right and left origins of replication from the Φ29 DNA replication machinery.
**G85**	Contains the DNA unit for the expression of Φ29 DNA polymerase. Transcription is regulated by a T7 promoter and vsv terminator sequences.
**G95**	Contains the DNA sequence for the expression of DNAP and TP. Each protein expression is independently regulated by a T7 promoter and a terminator sequence. DNAP unit uses a vsv terminator. TP unit utilizes a T7 terminator sequence. The entire CDS encoding for DNAP and TP is placed in between right and left origins of replication from the Φ29 DNA replication machinery.

Supplementary Table 6. Transitions of the MS/MS measurements for the proteolytic peptides of the indicated proteins.

Protein	Compound name	Precursor ion *(m/z)*	Product ion *(m/z)*	Collision energy (eV)	Accelerator voltage (eV)	Ion name
PSSA	DLQSIADYPVK.light	624.8272	805.4454	20.4	4	y7
PSSA	DLQSIADYPVK.light	624.8272	692.3614	20.4	4	y6
PSSA	DLQSIADYPVK.light	624.8272	506.2973	20.4	4	y4
PSSA	DLQSIADYPVK.light	624.8272	343.2340	20.4	4	y3
PSSA	DLQSIADYPVK.light	624.8272	357.1769	20.4	4	b3
PSSA. QconCAT	DLQSIADYPVK.heavy	631.3079	813.4217	20.4	4	y7
PSSA. QconCAT	DLQSIADYPVK.heavy	631.3079	699.3406	20.4	4	y6
PSSA. QconCAT	DLQSIADYPVK.heavy	631.3079	511.2825	20.4	4	y4
PSSA. QconCAT	DLQSIADYPVK.heavy	631.3079	347.2221	20.4	4	y3
PSSA. QconCAT	DLQSIADYPVK.heavy	631.3079	361.1650	20.4	4	b3
YFP	FEGDTLVNR.light	525.7644	903.4530	17.3	4	y8
YFP	FEGDTLVNR.light	525.7644	774.4104	17.3	4	y7
YFP	FEGDTLVNR.light	525.7644	717.3890	17.3	4	y6
YFP	FEGDTLVNR.light	525.7644	602.3620	17.3	4	y5
YFP	FEGDTLVNR.light	525.7644	501.3144	17.3	4	y4
YFP	FEGDTLVNR.light	525.7644	449.1667	17.3	4	b4
YFP. QconCAT	FEGDTLVNR.heavy	532.2451	915.4175	17.3	4	y8
YFP. QconCAT	FEGDTLVNR.heavy	532.2451	785.3778	17.3	4	y7
YFP. QconCAT	FEGDTLVNR.heavy	532.2451	727.3593	17.3	4	y6
YFP. QconCAT	FEGDTLVNR.heavy	532.2451	611.3354	17.3	4	y5
YFP. QconCAT	FEGDTLVNR.heavy	532.2451	509.2906	17.3	4	y4
YFP. QconCAT	FEGDTLVNR.heavy	532.2451	453.1548	17.3	4	b4
Ribosomal protein S4 (YFP quantification)	LSDYGVQLR.light	525.7826	850.4417	17.3	4	y7
Ribosomal protein S4 (YFP quantification)	LSDYGVQLR.light	525.7826	735.4148	17.3	4	y6
Ribosomal protein S4 (YFP quantification)	LSDYGVQLR.light	525.7826	572.3515	17.3	4	y5
Ribosomal protein S4 (YFP quantification)	LSDYGVQLR.light	525.7826	635.3035	17.3	4	b6
Ribosomal protein S4. QconCAT (YFP quantification)	LSDYGVQLR.heavy	525.7826	861.4091	17.3	4	y7
Ribosomal protein S4. QconCAT (YFP quantification)	LSDYGVQLR.heavy	525.7826	745.3851	17.3	4	y6
Ribosomal protein S4. QconCAT (YFP quantification)	LSDYGVQLR.heavy	525.7826	581.3248	17.3	4	y5
Ribosomal protein S4. QconCAT (YFP quantification)	LSDYGVQLR.heavy	525.7826	641.2857	17.3	4	b6
Ribosomal protein L6 (YFP quantification)	APVVVPAGVDVK.light	575.8452	883.5247	18.9	4	y9
Ribosomal protein L6 (YFP quantification)	APVVVPAGVDVK.light	575.8452	784.4563	18.9	4	y8
Ribosomal protein L6 (YFP quantification)	APVVVPAGVDVK.light	575.8452	685.3879	18.9	4	y7

Ribosomal protein L6 (YFP quantification)	APVVVPAGVDVK.light	575.8452	268.1656	18.9	4	b3
Ribosomal protein L6 (YFP quantification)	APVVVPAGVDVK.light	575.8452	367.2340	18.9	4	b4
Ribosomal protein L6 (YFP quantification)	APVVVPAGVDVK.light	575.8452	466.3024	18.9	4	b5
Ribosomal protein L6. QconCAT (YFP quantification)	APVVVPAGVDVK.heavy	582.3259	893.4951	18.9	4	y9
Ribosomal protein L6. QconCAT (YFP quantification)	APVVVPAGVDVK.heavy	582.3259	793.4296	18.9	4	y8
Ribosomal protein L6. QconCAT (YFP quantification)	APVVVPAGVDVK.heavy	582.3259	693.3642	18.9	4	y7
Ribosomal protein L6. QconCAT (YFP quantification)	APVVVPAGVDVK.heavy	582.3259	271.1567	18.9	4	b3
Ribosomal protein L6. QconCAT (YFP quantification)	APVVVPAGVDVK.heavy	582.3259	371.2221	18.9	4	b4
Ribosomal protein L6. QconCAT (YFP quantification)	APVVVPAGVDVK.heavy	582.3259	471.2876	18.9	4	b5
Ribosomal protein S1 (PSSA quantification)	GVVVAIDK.light	400.7475	644.3978	13.4	4	y6
Ribosomal protein S1 (PSSA quantification)	GVVVAIDK.light	400.7475	545.3293	13.4	4	y5
Ribosomal protein S1 (PSSA quantification)	GVVVAIDK.light	400.7475	446.2609	13.4	4	y4
Ribosomal protein S1 (PSSA quantification)	GVVVAIDK.light	400.7475	426.2711	13.4	4	b5
Ribosomal protein S1. QconCAT (PSSA quantification)	GVVVAIDK.heavy	405.234	651.3770	13.4	4	y6
Ribosomal protein S1. QconCAT (PSSA quantification)	GVVVAIDK.heavy	405.234	551.3115	13.4	4	y5
Ribosomal protein S1. QconCAT (PSSA quantification)	GVVVAIDK.heavy	405.234	451.2461	13.4	4	y4
Ribosomal protein S1. QconCAT (PSSA quantification)	GVVVAIDK.heavy	405.234	431.2563	13.4	4	b5
Ribosomal protein L1 (PSSA quantification)	VVGQLGQVLGPR.light	611.8670	1024.5898	20	4	y10
Ribosomal protein L1 (PSSA quantification)	VVGQLGQVLGPR.light	611.8670	839.5098	20	4	y8
Ribosomal protein L1 (PSSA quantification)	VVGQLGQVLGPR.light	611.8670	726.4257	20	4	y7
Ribosomal protein L1 (PSSA quantification)	VVGQLGQVLGPR.light	611.8670	442.2772	20	4	y4
Ribosomal protein L1 (PSSA quantification)	VVGQLGQVLGPR.light	611.8670	329.1932	20	4	y3
Ribosomal protein L1 (PSSA quantification)	VVGQLGQVLGPR.heavy	620.3418	1039.5453	20	4	y10
Ribosomal protein L1 (PSSA quantification)	VVGQLGQVLGPR.heavy	620.3418	851.4742	20	4	y8
Ribosomal protein L1 (PSSA quantification)	VVGQLGQVLGPR.heavy	620.3418	737.3931	20	4	y7
Ribosomal protein L1 (PSSA quantification)	VVGQLGQVLGPR.heavy	620.3418	449.2565	20	4	y4
Ribosomal protein L1 (PSSA quantification)	VVGQLGQVLGPR.heavy	620.3418	335.1754	20	4	y3
Ribosomal protein S4 (PSSA quantification)	AALELAEQR.light	500.7747	745.3839	16.5	4	y6
Ribosomal protein S4 (PSSA quantification)	AALELAEQR.light	500.7747	616.3413	16.5	4	y5

Ribosomal protein S4 (PSSA quantification)	AALELAEQR.light	500.7747	503.2572	16.5	4	y4
Ribosomal protein S4 (PSSA quantification)	AALELAEQR.light	500.7747	432.2201	16.5	4	y3
Ribosomal protein S4 (PSSA quantification)	AALELAEQR.light	500.7747	385.2082	16.5	4	b4
Ribosomal protein S4 (PSSA quantification)	AALELAEQR.heavy	507.2555	755.3542	16.5	4	y6
Ribosomal protein S4 (PSSA quantification)	AALELAEQR.heavy	507.2555	625.3146	16.5	4	y5
Ribosomal protein S4 (PSSA quantification)	AALELAEQR.heavy	507.2555	511.2335	16.5	4	y4
Ribosomal protein S4 (PSSA quantification)	AALELAEQR.heavy	507.2555	439.1994	16.5	4	y3
Ribosomal protein S4 (PSSA quantification)	AALELAEQR.heavy	507.2555	389.1963	16.5	4	b4

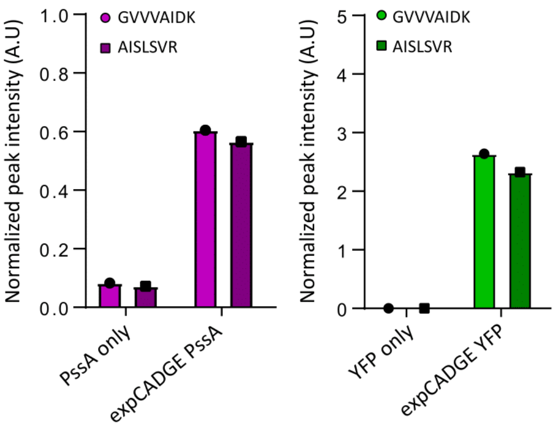

Fig S1. Relative protein quantification from bulk reactions with and without expCADGE. The maximum peak intensity of each POI proteolytic peptide was normalized to the maximum peak intensity of one of the two S1 ribosomal protein signature peptides (GVVVAIDK or AISLSVR) present in PURE system.

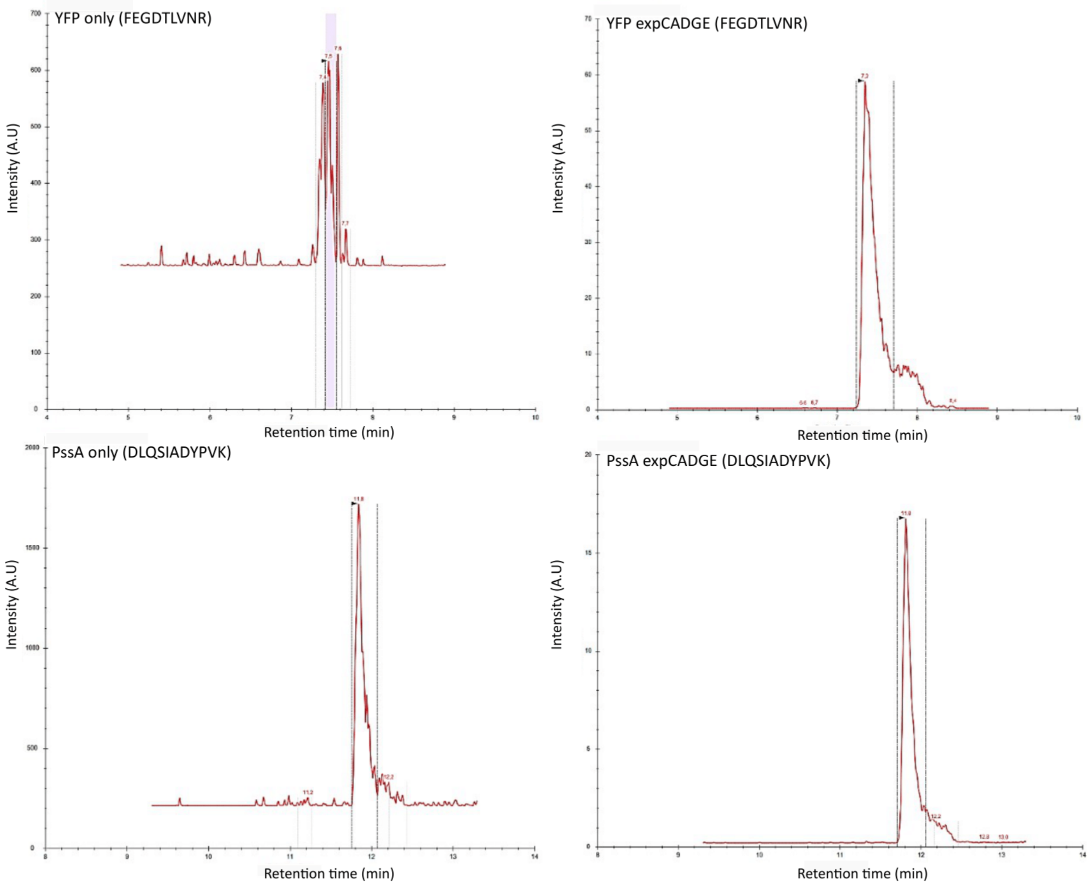

Fig S2. LC-MS raw data peaks of the YFP and PssA proteolytic peptides used for relative quantification.

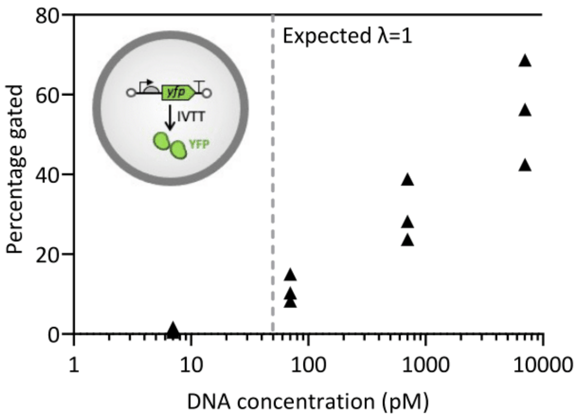

Fig S3. Effect of DNA concentration on YFP protein expression without gene amplification. Fluorescence from individual liposomes was analyzed by flow cytometry. The vertical dashed line indicates the DNA concentration that theoretically corresponds to λ = 1 if one assumes a monodisperse population of liposomes with a diameter of 4 µm and a random (Poisson) partitioning of DNA.

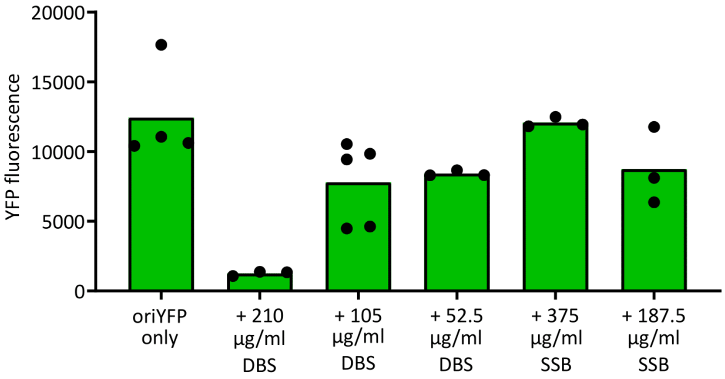

Fig S4. End-point YFP fluorescence measurements from ori-*yfp* bulk IVTT reactions. Protein expression can be inhibited under high DSB concentrations (210 µg/ml).

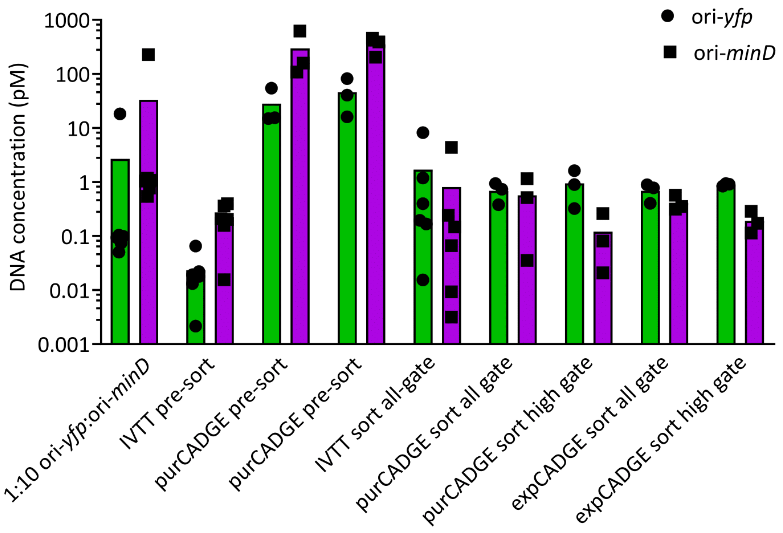

Fig S5. Individual qPCR data from Figure 4h. Enrichment of ori-*yfp* over ori-*minD*. Each symbol represents a biological repeat. 'IVTT' indicates a reaction without DNA replication.

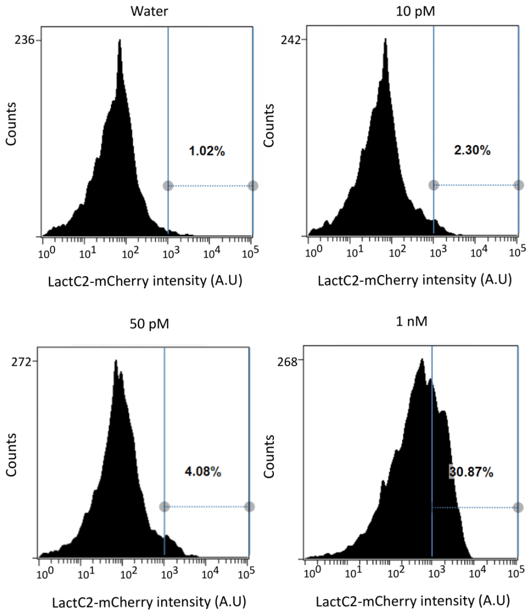

Fig S6. Raw FACS data of liposome samples with appended gating line as used in Figure 5c,d.

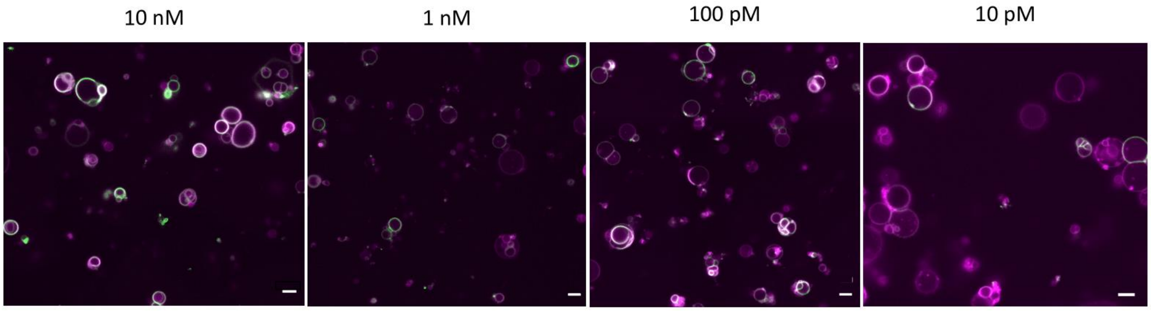

Fig S7. In-liposome expression of ori-*pssA* under different DNA concentrations (10 nM to 10 pM) without DNA replication. Liposome membrane dye (Texas-red) is colored in magenta and PS binding protein LactC2-eGFP is colored in green. Overlay of the two colors is displayed in white. Lowering the concentration of *pssA* gene reduces the number of liposomes with membrane-recruited LactC2-eGFP. Scale bars are 5 µm.

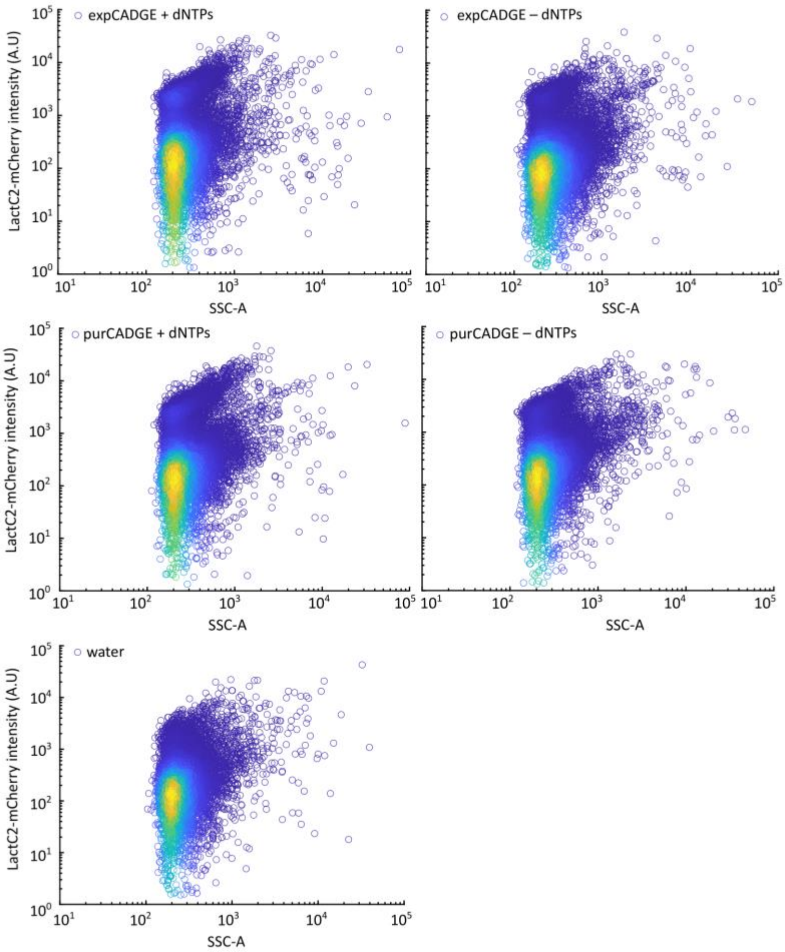

Fig S8. FACS data of liposome samples analyzed in Figure 5g.

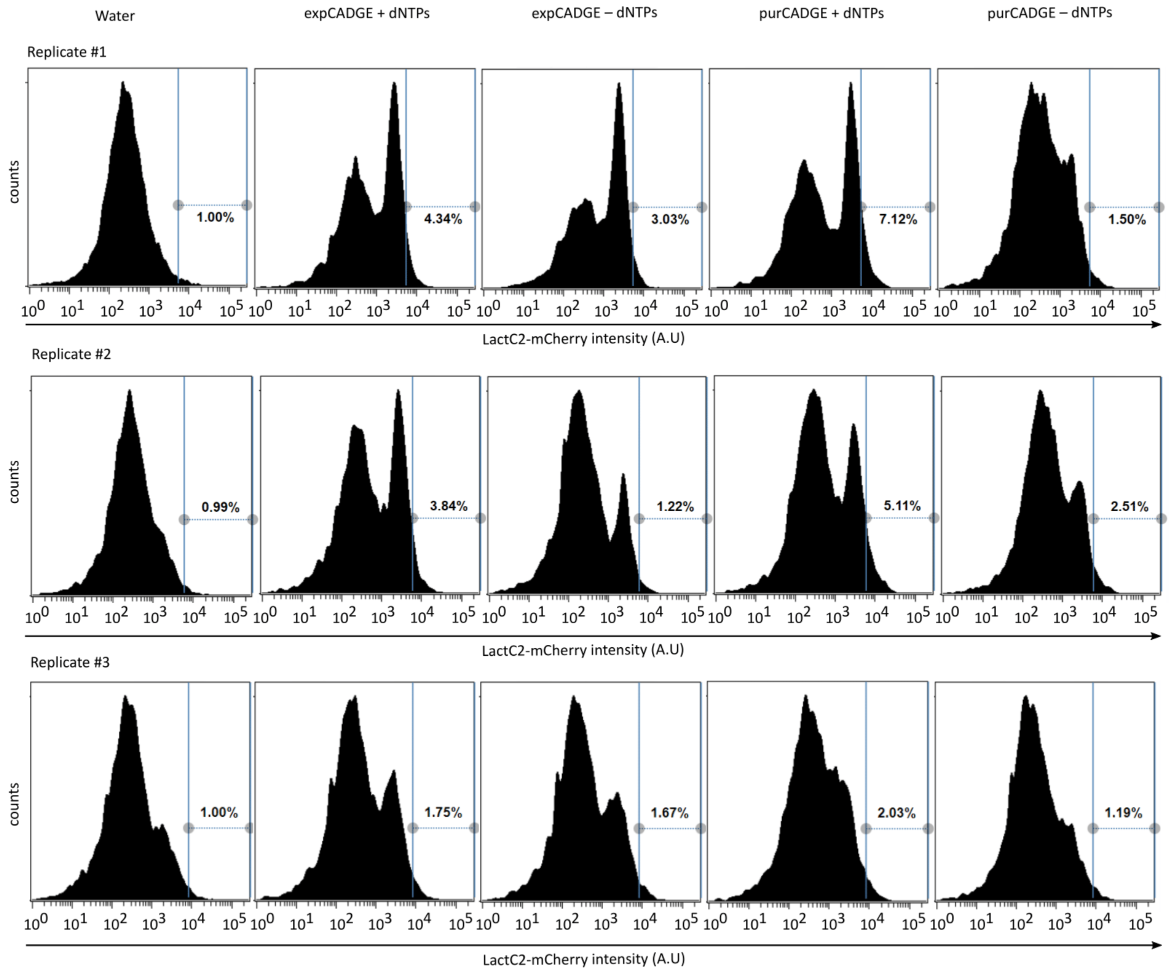

Fig S9. Liposomes FACS data and gating strategy for Figure 5h.

Figure 2

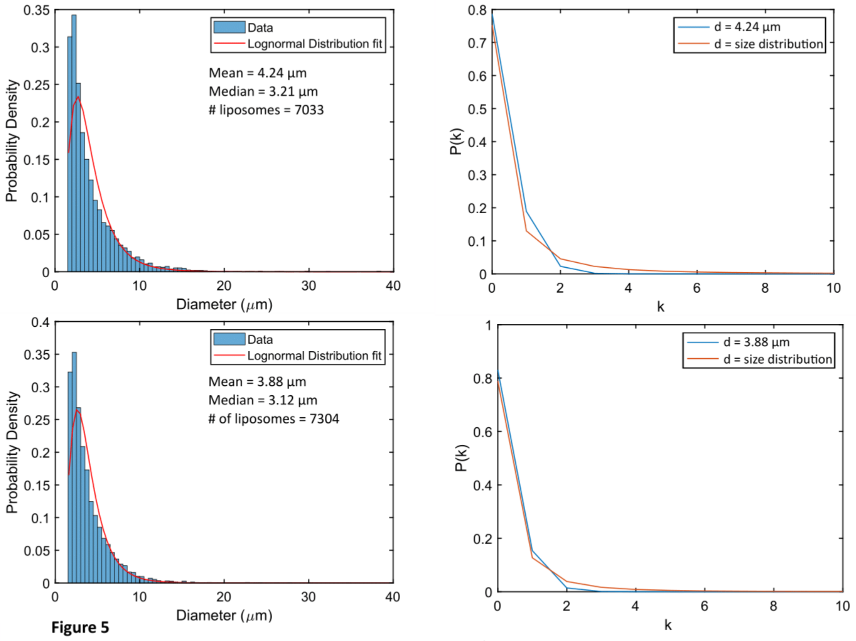

Figure 5

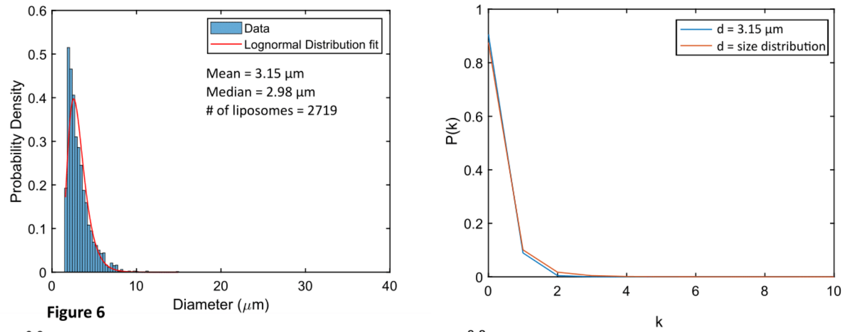

Figure 6

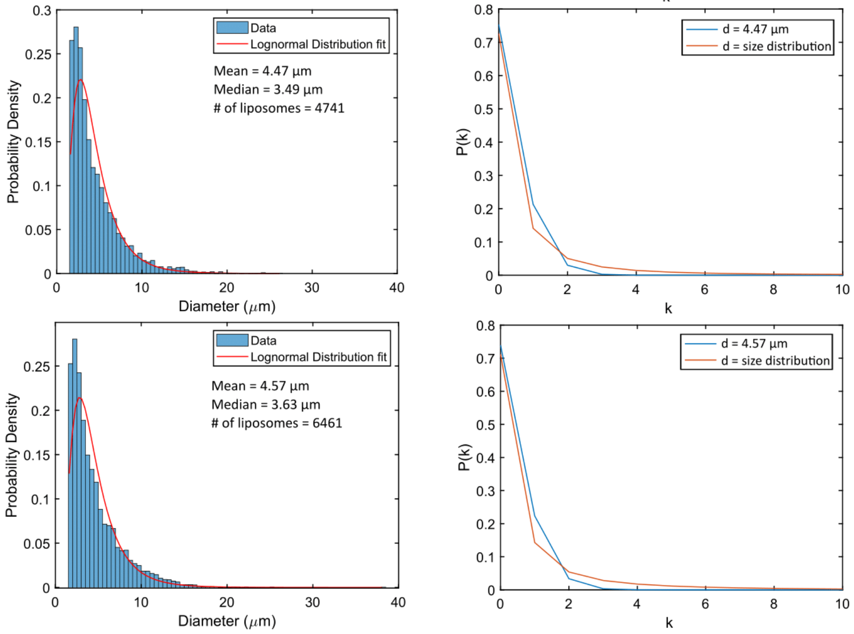

Fig S10. Quantitative analysis of liposome size distribution and DNA occupancy. Histograms of liposome sizes for different samples (left). The mean and median diameter values, as well as the number of liposomes analyzed per sample are appended on the graphs. The distributions were fitted with a lognormal function (red curves). The probability of having *k* DNA molecules on average per liposome was calculated by assuming a fixed diameter (distribution mean, in blue) or from the actual size distribution in the corresponding sample (in red).

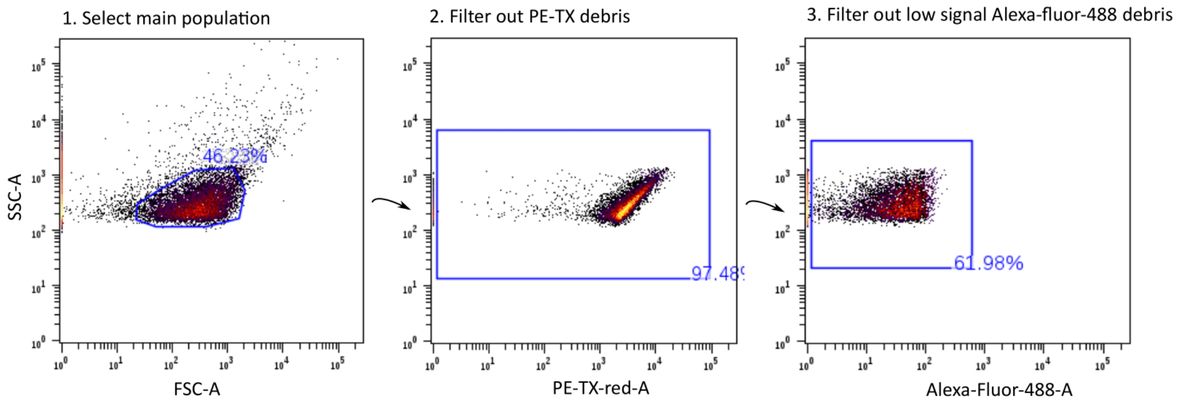

Fig S11. Data processing for FACS data. Application of the filtering gate to remove liposomal debris in PssA expressing-liposomes.
